# Clinical Delivery of Therapeutic Agents Based on Metals

**DOI:** 10.1155/MBD.1997.133

**Published:** 1997

**Authors:** John Fox

**Affiliations:** Shire Pharmaceuticals Plc. Planning Department East Anton Andover SP10 5RG UK

## Abstract

Metals have been used in clinical practice for hundreds of years and for a variety of indications. Although potent agents whose activity may be adapted by manipulation of their chemistry and that of associated ligands, their use has been limited by toxic effects. There is now a burgeoning series of delivery technologies available which may be adapted to the administration of metal based drugs. Together with greater understanding of metal chemistry and their mechanisms of action in disease processes, there is an opportunity to increase the use of metals in medicine by targeting their action more effectively to the therapeutic site and/or protecting the body from toxic effects.


### CLINICAL DELIVERY OF THERAPEUTIC AGENTS BASED ON METALS

John Fox

Shire Pharmaceuticals Plc., Planning Department,
East Anton, Andover, SP10 5RG, UK

### Abstract

Metals have been used in clinical practice for hundreds of years and for a variety of indications. Although potent agents whose activity may be adapted by manipulation of their chemistry and that of associated ligands, their use has been limited by toxic effects. There is now a burgeoning series of delivery technologies available which may be adapted to the administration of metal based drugs. Together with greater understanding of metal chemistry and their mechanisms of action in disease processes, there is an opportunity to increase the use of metals in medicine by targeting their action more effectively to the therapeutic site and/or protecting the body from toxic effects.

### Introduction

Metals have been used in medicine for hundreds of years. Most common delivery methods were via the oral route and topical application. Indications ranged from the treatment of syphilis with arsenic or mercury to Chinese herbal remedies which contained lead, mercury and arsenic among others [1]. Populations have also been exposed to metals through their use in agriculture, for instance the use of mercury as a fungicide on seed corn [2] which led to many deaths in the early 1980's in Iraq when food shortages led to the use of seed grain to make bread despite Government warnings.

There are still many areas of clinical application where metals make a significant contribution. Examples would include the use of platinum (cancer chemotherapy), mercury (dental amalgams), gold (Rheumatoid Arthritis), Silver (wound healing), Technetium (imaging), Iron (supplements) and so on. A general theme evident in the use of metals in medicine throughout history is the balance of efficacy with respect to the target biochemical system and the potential for immediate or long term toxicity. This paper seeks to explore the delivery means that are currently available or in development that may assist in retaining activity at the target site while reducing the toxicity of metal-based therapeutics. Successful formulations would allow the potency of metals to be deployed in a much wider range of indications and at an earlier stage in those indications where use is currently limited by toxicity.

### Discussion

There are several issues which influence the use of metals in appropriate therapeutic areas. They tend to have a high potency, but their effects may be relatively unspecific. The pharmacological activity of metals is essentially a function of their chemistry but structure activity relationships may be influenced by factors such as available ligands or oxidation state.

Toxicity may be acute, as in the cytotoxic nature of the cisplatinum compounds used as chemotherapeutic agents in the treatment of some cancers, or long term. Long term toxicity commonly arises due to accumulation over time as in the case of osteomalacia due to the accumulation of aluminium by patients with end stage renal disease. This toxicity potential has severe implications for the development of products which would receive a Marketing Authorisation from regulatory bodies. Regulatory Authorities look for clear demonstration of quality, safety and efficacy in Product Licence Applications. Assuming the quality of product is appropriate - no simple matter in a highly regulated manufacturing environment - a product is approvable only if it has advantages in efficacy and/or safety over existing treatments, pharmaceutical or otherwise. Cost is not a basis for approval or rejection, although pricing will have an influence on the extent of use of a licensed product.

The use of many metal based drugs is limited by their toxicity to severe diseases or other conditions that are unresponsive to normal management regimens. In these cases the risk:benefit ratio becomes acceptable because there are few or no alternative means of active treatment. This indicates that the challenge faced by those developing new metal based drugs is to target the active principle more effectively and/or to mask the toxic properties of the preparation until it reaches its site of action.

There are many different routes of delivery of drugs to the body. These include:

Oral
Sub-cutaneous
Parenteral
Including implants/pumps
Transdermal
Patches, gels
Passive; Active (ie Iontophoresis)
Mucosal
Buccal, nasal, rectal, vaginal
Inhalation
Intrathecal
Intracapsular
Opthalmic

Strategies for the delivery of metals could involve the use of carrier molecules as targeting vehicles or the masking of a potentially toxic moiety. Much work has been done on the use of biodegradable polymers as transport agents and long-acting enzyme preparations have been developed using polylactide-coglycolide (PLGA) and polyethylene glycol polymers[3].

Issues to be addressed for this type of formulation largely relate to the technical challenges associated with combining the active agent with its carrier, delivery of the combination and presentation of the metal in its active form at the target site. Since there may still be some systemic exposure to the unprotected metal a risk of residual toxicity remains. The validity of such an approach will depend on the target site to be reached, likely carriers and the chemistry of the metal and potential ligands.

An alternative strategyis to encase the metal complex within an external structure such as a liposome, constructed of polar lipid molecules, or proteinoids, hollow microspheres of condensed amino-acids. This strategy could enhance absorption, potentially reduce toxicity and protect the active moiety from deactivation before it reached the target site. Wrapping technologies are able to protect the active agent through the processes of absorption and distribution. They involve embedding the drug within a matrix whose physicochemical properties can be adjusted to suit the properties of the compound and the chosen delivery route. At present the only liposome-based marketed products are indicated for systemic fungal and mycobacterium infections and for dermatological use. However products are in development for use as cancer chemotherapeutic agents.

An alternative means of protecting the active molecule could make use of a pro-drug approach, whereby biochemical processes within the body modify the formulation to release the active form. Again, challenges to be overcome would most likely arise from technical issues with respect to initial manufacture of a reproducible formulation and consistent release of the therapeutic agent at the site of action.

Several of the established routes of administration could be used for the above strategies. Oral formulations represent the majority of novel drug delivery systems[3] and carrier based strategies are available to increase the bioavailability of high molecular weight compounds or complexes. The use of the oral route can provide opportunities to tailor the kinetics of the product, including the onset time of drug release, or if appropriate the site of absorption within the gastrointestinal tract.

Inhalation technology is now able to deliver therapeutic levels of high molecular weight compounds at low inspiration rates using propellant-free devices. The deep lung has a large surface area for drug delivery and constitutes an opportunity for rapid delivery of compounds capable of being formulated in dry powder form.

Targeted delivery could include systems such as the use of viral vectors or monoclonal antibodies to ensure the active agent is delivered preferentially to the target site in sufficient amounts while reducing the potential for side effects. For products where continuous delivery is desirable a range of implantable polymers, hydrogels and pumps are available. Novel polymers and ceramic particles are under development as controlled release media for injectable products. For all these technologies, consideration should be given to the advantage of the new delivery in clinical practice which will involve comparison of the efficacy and safety profiles against accepted treatments.

In summary, the challenge for the future development of metals in clinical practice is to improve current means of delivery where metals are of known benefit, to identify additional therapeutic uses of metals in medicine and to link potential uses of metals to enabling technologies. The recent development of innovative means of delivery for pharmaceuticals of various molecular weights and physiochemical characteristics has introduced an opportunity to expand the use of metals in medicine. By imaginative construction of desired product profiles and close collaboration between chemists, physicians and delivery technology specialists it will be feasible to construct formulations that will make best use of the potency of metals while improving their safety profile.

### References

1.*Acta. Paediatr. Sin.*, 1993, **34,** 181-190
2.*Handbook on the Toxicology of Metals*, 1986, **2**, 2nd edn.
(Eds. Friberg, L., Nordberg, G.F., and Vouk, V.B.) ,
3.*European Market for Drug Delivery Systems*
Report No 1860; Frost and Sullivan (Pubs.)

